# Experiences of rural women with damages resulting from an earthquake in Iran: a qualitative study

**DOI:** 10.1186/s12889-020-08752-z

**Published:** 2020-05-06

**Authors:** Javad Yoosefi Lebni, Farhad Khorami, Farbod Ebadi Fard Azar, Bahar Khosravi, Hossein Safari, Arash Ziapour

**Affiliations:** 1 Health Education and Health Promotion, School of Health, Iran University of Medical Sciences, Tehran, Iran; 2 Master of Clinical Psychology, Islamic Azad University, Kermanshah Branch, Kermanshah, Iran; 3 Department of Education and Health Promotion, School of Health, Iran University of Medical Sciences, Tehran, Iran; 4 Students Research Committee, Kermanshah University of Medical Sciences, Kermanshah, Iran; 5 Health Promotion Research Center, Iran University of Medical Sciences, Tehran, Iran; 6 Health Education and Health Promotion, Health Institute, Kermanshah University of Medical Sciences, Kermanshah, Iran

**Keywords:** Earthquake, Rural women, Qualitative study, Iran

## Abstract

**Background:**

Women, with more vulnerabilities and less access to resources, are often seen as victims of natural disasters. Therefore, the present study aimed to investigate the experiences of rural women with damages resulting from an earthquake in Iran.

**Methods:**

In this research, a qualitative approach, as well as the conventional content analysis was employed. The study population consisted of rural women residing in the earthquake-stricken areas of Sarpol-e Zahab and Salas-e Babajani counties in Kermanshah Province, Iran. Semi-structured interviews were used for data collection. Moreover, sampling was purposeful, theoretical saturation was achieved by conducting 22 interviews, and the data analysis process was performed according to the steps proposed by Graneheim and Lundman. For the strength and transferability of the research, Lincoln and Guba’s Evaluative Criteria were used.

**Results:**

There were seven categories regarding the experiences of rural women after the earthquakes including neglecting the health needs; tension in the family and marital relations; gender inequality in the provision of assistance; feeling insecure; ignoring the ruling culture of the region; concealing needs for fear of stigmatization, and incoherent mourning as well as two categories regarding their reactions to and interaction with the earthquake consequences including positive and negative interactions.

**Conclusions:**

Paying more attention to the needs of rural women, taking the culture governing the village into account at the time of service delivery, and helping them with positive adaptations are some indispensable measures that should be taken.

## Background

Earthquakes, as the second deadliest natural disaster, have always existed throughout the life of the planet. These are unpredictable and out-of-control, which can cause extensive destruction, threaten many lives [1–4], and can leave profound and lasting effects on the minds and development of human beings [5]. As reported by the World Health Organization (WHO), over the past century alone, 1150 fatal earthquakes have been happened in 75 countries worldwide [6]. Although most natural disasters occur in developing countries, they are not sufficiently equipped with the necessary resources and structures to provide survivors with proper services [7]. People in developed countries are less affected by mental problems associated with earthquakes due to the availability of social protection systems in these countries [8]. Iran, as a developing country, records the severity of earthquakes with titanic magnitudes every four years, whereby 97 and 79% of rural and urban units are demolished, respectively. For instance, over 170 thousand people have lost their lives in major earthquakes that occurred in the Iranian cities such as Torbat-e Heydarieh, Kerman, Bam, Shirvan, Larijan, Rudbar, Tabas, Ahar, and Varzaghan [9]. As another example, a 7.3-magnitude earthquake rocked the Kermanshah Province at 9:48 p.m. on Sunday, November 21, 2012, resulting in massive destruction and casualties. According to Iran’s Forensic Medicine Organization, the death toll stood at 620, as well as leaving 9388 injured and 70,000 homeless [10].

Recent studies have shown that the damages caused by natural disasters are not equitable among different groups of people [11]. In the events of natural disasters, the pre-existing hierarchies and inequalities in access to resources, capabilities, and opportunities, make the certain groups more vulnerable to more suffering [12] Recent research has shown the unique effects of natural disasters and earthquakes on vulnerable groups such as women, children and ethnic minorities [13, 14]. Moreover, the results of sociological and ethnographic research have also revealed that gender and social inequalities are exacerbated by natural disasters [15].

One’s reaction to natural disasters is heavily influenced by his/her cultural, social, economic and political backgrounds [16, 17]. Hence, natural disasters are perceived differently by men and women [13, 18].

Not to mention, women, with more vulnerabilities and less access to resources [19, 20], are often seen as the victims of natural disasters [21].

The study of Anwar et al. (2011) and Demirchyan et al. (2014) on Pakistani women stricken by earthquakes concluded that those women had multiple health problems and notable danger of depression and anxiety [4, 22]. Doris et al. (2016) explored that the female survivors of the Haiti earthquake experienced various physical and psychological problems and also a lot of violence from their husbands or boyfriends. However, 60–78% of women did not report any case of violence [23]. The study of Chan and Zhang (2011) on women troubled by an earthquake in Sichuan, China, revealed that all kinds of domestic violence, ranging from sexual to physical, was on the rise between couples after the earthquake, thereby disturbing the mental and physical performance of victims [24]. In a few studies conducted by Liu et al. (2012) and Qu et al. (2012) on rural women stricken by earthquakes in China, the results revealed that 52.2% of participants had psychiatric disorders, and widows and those who witnessed their loved ones dying had more psychiatric disorders. Besides, the women working outside villages experienced fewer psychiatric disorders than the unemployed women living in these villages [25, 26]. Among women, rural women are the weakest groups because of the traditional social and cultural contexts that govern their villages, who can suffer from numerous physical and psychological damages as a result of natural disasters such as earthquakes.

Most studies conducted on earthquakes are quantitative and empirical, and a few previous studies have addressed the experiences of rural female survivors qualitatively. It should be noted that qualitative studies have a unique ability to understand phenomena and to describe life experiences, human interpretations and perceptions in the same cultural and social context [27]. Hence, the qualitative study of rural women’s experiences of earthquakes can provide health workers and policymakers with extensive information and necessary knowledge to appropriately respond to earthquakes. Therefore, the present study aimed to investigate the experiences of rural women with damages resulting from an earthquake in Iran.

## Methods

In this research, a qualitative approach and the conventional content analysis were employed to explain the experiences of rural women with damages resulting from an earthquake in Iran. Qualitative content analysis is one of the several research methods and a robust process that is used to analyze textual data and is intended to start the introductory meanings of the concept. It is also a systematic categorization and coding method. The main goal of this method is to satisfactorily understand the phenomenon being investigated [28].

Moreover, the study population consisted of rural women residing in the earthquake-stricken areas of Sarpol-e Zahab and Salas-e Babajani counties in Kermanshah Province, Iran, who were there on the night that the earthquake rocked these two counties on November 12, 2017. Moreover, the inclusion criteria were consciousness, willingness to express their experiences of the earthquake, being female, residing in rural areas, ability to speak and willingness to participate in the research.

To commence the study, the required permits were obtained from the Vice Chancellery for the Department of Research and Technology at Kermanshah University of Medical Sciences. For data collection, semi-structured interviews were used. However, given that the researchers were natives to the earthquake-stricken areas and served there as volunteers, the observation method was also used. Furthermore, the interviews were commenced two months after the earthquake and lasted for four months. Besides, the interviews were conducted based on some general questions (Table 1). The interview guide was developed specifically for this study.

**Table 1:** Interview questions

1	Explain to us what happened after the earthquake?
2	What were your main problems after the earthquake? Please explain
3	Were your personality and behavior changed after the earthquake? Please Explain
4	What impact did the earthquake have on your family? Did it affect inter-family relationships? Please Explain.
5	Were your health needs met as a woman after the earthquake? Please Explain
6	How was the distribution of aid among the affected people? Were you satisfied with the services they provided for you? Please explain.
7	Could you easily access the services they provided for you?
8	How do you evaluate the overall status of different organizations in terms of the services they provided for you? What were the weaknesses of their services? Please explain.
9	How did you deal with the post-earthquake conditions? Please explain
10	Did any of your family die in the earthquake? If yes, please explain what you think of his death.
11	What were you doing to make it easier to cope with earthquake damages?

After asking the general questions that were the same for all participants, the following questions were developed concerning the answers that they provided. Any new code that emerged in the interview was attempted to be asked as a question in the subsequent interviews to check the codes repeatedly to find out whether or not other interviewees have had such an experience so that researchers can gain richer concepts.

During the interviews, given that the researchers used local language of the interviewees, they were asked to speak in their local language to express their experiences comfortably, thereby creating a sense of trust and willingness to participate in the study. The time and place of the interviews were decided by the samples, which were mostly held in container homes and tents without anyone accompanying them since it was likely that other people would prevent them from communicating their experiences to the researchers smoothly. The average duration of the interviews was 37 min. After each interview, the researchers began to analyze and encode the data to use them as a kind of guidance in subsequent interviews. Moreover, sampling was purposeful, and theoretical saturation was achieved by conducting 22 interviews and the main and sub-categories were formed. In qualitative research, the sampling criterion is the theoretical saturation, and whenever there is no new code in interviews, it shows that saturation is reached and the researchers can interrupt the interview process and no longer need to increase the sample size [29]. In fact, in an interview with the 18th participant, all the codes were repetitive and no new code was formed; however, the researchers performed four more interviews to make sure of this, and in the next four interviews, no new code was created, so the researchers concluded that continuing another interview is not necessary because they had reached the saturation and there was no new code.

In the present study, the data analysis process was performed according to the steps proposed by Graneheim and Lundman (2004). Additionally, the data analysis began simultaneous with data collection, and all interviews were recorded, and the contents were written verbatim. Besides, the entire material was reviewed several times to gain a correct understanding [28]. At the data preparation stage, to get acquainted with the data, the entire text was read in full and the researchers immersed themselves in understanding the contents of interviews. Bedsides, the researchers took general notes of their ideas of the interviews. Next, at the defining semantic units’ stage, the semantic units were identified and the primary codes were extracted. Then, at the coding, the text and classifying and developing themes and subthemes stages the codes were merged and classified according to their similarities and named under different sub-categories. Then, at identifying the main themes stage, several sub-categories fell into one category based on at least one common feature, which was the basic part of content analysis. It was also tried to have the most homogeneity within the categories and the most heterogeneity between the categories.

Since qualitative research has slightly different paradigmatic principles than quantitative research, the methods of evaluating and generalizing results in qualitative research are different from quantitative research [30]. Therefore, for the credibility and transferability of this study, the Guba and Lincoln criteria were used, which is one of the most common criteria for evaluating the quality and reviewing the principles and strategies of accreditation and generalizability in qualitative research [31]. After encoding and data analyses, the data were given to some samples to confirm their accuracy and to correct them if it is necessary (member Check). In addition, some parts of the interview texts along with codes and categories were sent to some psychologists and social workers to verify the accuracy of the data analysis (Checking by experts). Moreover, maximum diversity was used in sampling to provide more comprehensive and complete information (Diversity in samples), and direct quotations were also used.

To observe the ethical considerations, the objectives of the present study were explained to the target subjects, and they were assured that their information would be kept confidential. They were also assured that their data would be encoded and published in such a way that they could not be identified by any means. Moreover, they were given the right to interrupt the interview at any time.

In this research, different to most existing studies, the problems and needs of rural women after the earthquake were examined qualitatively, which can provide unique information and give us the needs and problems of rural women after the earthquake to prepare the grounds for intervention to improve their health condition. Similarly, since some of the authors of the article were indigenous to the studied areas and were working as a social worker there, they could establish a close relationship with the participants, could gain a comprehensive familiarity with the research field, and could get more complete and hands-on information from the research field for readers and, therefore, pave the grounds for further researches.

## Results

### Sociodemographic profiles

In Table 2, the demographic information of the samples is presented. As can be seen, the average age of the samples was 25 years old and most of them were married and illiterate. Besides, most of them were unemployed and lived in container homes.

**Table 2:** The demographic information of the samples

Variable	Dimension	Frequency
Age	Mean	25
	Minimum	15
	Maximum	41
Level of education	Illiterate	9
	High school education and below	8
	High school education and above	5
Marriage	Single	8
	Married	10
	Other	4
Occupation	Unemployed	14
	Employed	3
	Self-employed	5
Lodging	Tent	5
	Container home	11
	House	6

After encoding and analyzing the obtained data, there were seven categories regarding the experiences of rural women after the earthquakes, as well as two categories regarding their reactions and interaction with the earthquake consequences (Tables 3 and 4).

**Table 3:** The experiences of rural women stricken by earthquakes

Categories	Sub-categories
Neglecting the health needs	Inappropriate access to the bathroom, limited access to sanitary napkins and failure to change them, and lack of proper underwear
Tension in Family and Marital Relations	Emotional disturbance in the relationship, sexual dysfunction with the spouse, increased physical violence in the family, increased abusiveness by the people they know
Gender inequality in the provision of assistance	Prioritizing the demands of men over women and providing services and equipment based on demand rather than necessity
Feeling insecure	The entry of strangers into the traditional atmosphere of the village and fear of disturbance
Ignoring the ruling culture of the region	The incompatibility of the sent clothing and food with the native culture of the region, provision of health services such as baths and so on, regardless of the native culture of the region
Concealing needs for fear of stigmatization	Concealing needs for fear of stigmatization
Incoherent mourning	Longing for seeing the loved ones who died in the earthquake, and continued mourning for the loved ones

**Table 4:** Rural women’s reaction to earthquakes

Categories	Sub-categories
Positive Interactions	Getting closer to God, participating in group works, comparing the current circumstances with other worse conditions, sharing feelings and experiences, and heroizing the dead
Negative Interactions	Annoyance at God, phobic display of a greater disaster, aggression towards the others, isolation, and death wish

### Experiences of rural women stricken by earthquakes

Rural women are one of the most vulnerable groups against earthquake who can be affected by it in a different way than other groups. Below is a list of the experiences of earthquake-stricken rural women in Kermanshah

1. Neglecting the Health Needs: Women have their own health needs, which in critical situations such as natural disasters need to be addressed to avoid health problems. However, after the earthquake, the most important health needs of women, such as access to baths and toilets, underwear, and sanitary napkin were ignored in the studied areas, and most of the rural women were in terrible conditions. After the earthquake, less attention was paid to the health needs of women in rural areas.

“It was really hard to find a restroom in the first days of the earthquake and we couldn’t take a shower until two months after the earthquake”. (interviewee 3).

“After the earthquake, many women in the village got their periods because of the horrible conditions they went through, but without any access to sanitary napkins.” (interviewee 15).

“All of my clothes were buried under the debris, and after several weeks passing from the earthquake, I had only one dirty dress to wear.” (interviewee 8).

Despite the urgent need of earthquake-stricken rural women to sanitation stuff, this need was largely ignored, which could put their health at great risk and make the process of returning to normal life difficult.

2. Tension in Family and Marital Relations: The consequences of the earthquake were so extreme that the living conditions of survivors were influenced, thus possibly leading to tensions in the family and disruption of marital relations. As many of the participants stated, after the earthquake, there was tension in the family and their family relationships were not as strong as before, and the family atmosphere was affected by the earthquake, even the sexual relations between the couples.

“After the earthquake, the atmosphere at our home was not like before and was tense. Plus, I could not make emotional affairs with my husband.” (interviewee 11).

“Despite the passage of several months from the earthquake, nothing is like before, and I feel my husband and I are emotionally distant. We used to talk so much, but now he has changed into a different person.” (interviewee 8) “After the earthquake, I could not have sex with my husband for a few months. To be honest, I didn’t feel like it and he was not interested as well.” (interviewee 2). “After the earthquake, my husband was in a bad temper and shouted at me all the time. He was not like this before.” (interviewee 1). “After the earthquake, all family members were psychologically affected, we talk less and sometimes we fight together”. (interviewee 9).

Since the family can play an effective role in dealing with post-earthquake issues, tensions within the family can make the process of returning rural women to normal life more difficult.

3. Gender Inequality in the Provision of Assistance: Since the earthquake-stricken areas were among the deprived areas of Iran and still dominated by the traditional patriarchal atmosphere, women possessed a low socioeconomic status and did not find the chance and appropriate conditions to receive aid.

“Although questions were asked only to men and they talked only about family problems and their problems, no one asked us about our problems and needs.” (interviewee 21).

“The aid was given only to men, and only strong persons got more aid. Sad to say, women were the last to have the things which were left”. (interviewee 7).

“Our problems were not given much attention, they gave everything first to men and if there was something left, they gave it to us” (interviewee 9).

Reproduction of gender inequality after natural disasters can make conditions worse than before for women and bring about more limitations for them, making it difficult or even impossible to access the resources and assistance provided. Therefore, considering the needs of women and prioritizing their demands because of their circumstances can provide the basis for faster rehabilitation.

4. Feeling Insecure: After the earthquake, many Iranians volunteered to act spontaneously and went to the earthquake-stricken areas and stayed there for some time after the earthquake. Therefore, their presence disturbed the previous social atmosphere of villages, thereby creating a kind of insecurity in rural women.

“After the earthquake, many unknown people commute to/from our village, and we are too afraid to go outside.” (interviewee 15). “Most of the time, we just stay in our container homes and do not get out. Our village has become too packed with strangers, and we are afraid of verbal and physical harassment.” (interviewee 6). “I do not feel good about the messy conditions of the village, I used to go out more, but now I feel insecure.” (interviewee 12).

Therefore, despite the good intentions of people from elsewhere to support recovery by going tothe stricken areas to volunteer, the presence of a large number of volunteers and strangerscauses further disruption, and makes it harder for rural women’s lives to return to stability.

5. Concealing Needs for Fear of Stigmatization: Given the traditional social and cultural fabric governing the earthquake-stricken areas, many women were reluctant to express their desires to avoid stigmatization. Therefore, providing conditions for rural women to help them more easily express their needs and access provided services could help them to be less vulnerable to earthquake damages.

“After a few days passed from the earthquake, sanitary napkins were brought to villages. Although I knew that several women were in dire need of sanitary napkins, they were too shy and afraid to say anything”. (interviewee 17).

“I am the head of the family, and it’s been nearly eight years that I have lost my husband. So, after the earthquake, I didn’t have anyone to get the things we needed, and I was too shy and afraid. That is why I was the last to receive a tent.” (Interviewee 12).

“To be honest, I had a yeast infection and I was too shy to speak about it or ask for anything.” (Interviewee 11).

Therefore, despite the severe needs of rural women to various facilities and services, these needs were often ignored and no one was trying to address them, this issue puts more pressure on these women.

6. Ignoring the Ruling Culture of the Region: The earthquake rocked the Kurdish areas, where the cultural and social fabric is different from those in other areas of Iran. Unfortunately, ignoring this main factor at the time of aid provision caused some problems for relief efforts.

“After the earthquake, I had only one dress to wear for a month because the clothes they sent us did not fit our cultural fabric, so we couldn’t wear them.” (Interviewee 16).

“We couldn’t wear most of the clothes and shoes that people had sent us, and once I wore one of those clothes, it was too noticeable. Thus, I was forced to take it off.” (Interviewee 18).

“In the first days after the incident, we couldn’t eat some kinds of foods that people had sent us, such as a baguette.” (interviewee 12). “Several days after the earthquake, several bathrooms were built in the village, but many other women including myself were too shy to use them at all.” (Interviewee 1).

One of the main pillars of intervention in any society is the recognition of its culture and customs so that without sufficient knowledge and understanding of its culture, it is not possible to identify their needs properly or even the assistance and services provided may be contrary to their customs so that the locals will be reluctant to receive them. Since the people living in the earthquake-stricken areas were Kurds and had a different social and cultural structure than other geographical areas of Iran, ignoring this different structure in many cases made the aids sent back without any use.

7. Incoherent Mourning: In earthquake-stricken areas, people are buried after holding ritual and funeral ceremonies that may take several days and families may feel bad if this is not properly done. Since earthquakes take place very quickly and bring about horrible consequences, many families are forced to bury their loved ones quickly, thereby causing dissatisfaction in them.

“My daughter was trapped under the rubble of our house, and when she was pulled out dead from the rubble, I was in the hospital. Sad to say, I was too late to arrive at her burial and couldn’t see her. I still get angry about why I couldn’t see her for the last time.” (Interviewee 4).

“When my father died on the night of the earthquake, we buried him in the morning, and there were almost no relatives present at his funeral. However, now after two months passing from his death, everyone offers condolences and we all wish he had died normally so his death wouldn’t make us so miserable.” (Interviewee 11).

Since the people who died from the earthquake were buried in different conditions, their families were unhappy with the situation and in fact, had a guilty conscience for not being able to hold a better funeral ceremony for them.

### Rural Women’s Reaction to Earthquakes

After the earthquake, rural women selected specific strategies to cope with the earthquake which fall into two categories: positive and negative interactions.

### Positive interactions

Positive Interactions: After the earthquake, some rural women tried to cope with the earthquake through a series of strategies and in fact found some kind of positive adaptation to the earthquake, which made them more relaxed and able to return to normal life. Positive interactions refers to the measures and approaches adopted by rural women, whereby they could better adapt themselves to the situation.

1. Getting Closer to God: In many cases, earthquakes are seen as an occurrence based on God’s will. Therefore, this natural occurrence can make changes in the connection between people and God, thereby making them closer to God.

“The earthquake reminds me of God and shows how weak we are. After the earthquake, I pray and try to be a better person.” (Interviewee 21).

“After the earthquake, I do not know if I were afraid or it was because of anything else that I swore to God to say my prayers and to think of God more. I used to pray less, but now I say my prayers every day because it gives me peace of mind.” (Interviewee 20).

To do this, rural women sought to find a source of comfort for themselves by doing things such as praying and thus calmed themselves down this way.

2. Participation in Group Work: After the earthquake, the women who were residing in less affected areas tried to help others by baking bread or doing some other voluntary services for other villages.

“Our village was ruined in the earthquake, but luckily nobody died. So, other women and I tried to bake bread for them and help with humanitarian aid. By doing so, I felt much better and thoughtless about the unpleasant conditions we were involved in.” (Interviewee 2).

“Other women and I gathered our extra Kurdish dresses and sent them to the areas where there was more destruction.” (Interviewee 15).

Participating in teamwork and being with other disadvantaged people made women less likely to think about their problems and this was a positive strategy to deal with this natural disaster.

3. Comparing the Current Circumstances with Other Worse Conditions: The lives of all residents in Kermanshah were affected by the earthquake, which imposed more financial losses on some people. In some cases, the earthquake survivors tried to compare their own circumstances with the others who were more damaged.

“Although our house is ruined, it is a blessing that at least my children are alive. Sad to say, my sister, lost her kid and her house was completely destroyed, too.” (Interviewee 10).

“One of my sons was killed in the earthquake, and in spite of all the trouble that the earthquake has caused us, it’s a blessing that at least two of my kids and my husband are still healthy and I am not like my neighbor who lost his whole family in the earthquake.” (Interviewee 18).

One important psychological strategy that women chose to cope with their worse conditions in order to make it easier to tolerate awful conditions was comparing themselves to others who were far worse off.

4. Sharing Feelings and Experiences: After the earthquake, the survivors were passionate about describing what the earthquake and its outcomes were like, thereby relieving themselves to some extent.

“After the earthquake, our only delight is sitting with other women in the village and thinking about the night when the earthquake occurred.” (Interviewee 13).

“These days, the only thing that amuses me is sitting with other women in the village and speaking about the night when the earthquake occurred and its consequences. The good thing is that at least we understand each other.” (Interviewee 15).

In fact, sharing emotions and memories with other women makes rural women more likely to cope with post-earthquake events.

5. Heroizing the Dead: Many of the women who lost their relatives in the earthquake were proud of them and believed that their relatives would have God’s mercy on their souls because they lost their lives in the earthquake, thus making it easier for them to come to terms with the death of their loved ones more easily.

“At first, I became desperately unhappy when my son died, but when I thought with myself that he was killed by the earthquake and God would forgive his sins for sure, I became calm and relieved”. (interviewee 2).

“I am thankful to God that at least my innocent girl is dead in the earthquake, which is one of the best deaths because God himself will bless them. I believe that they are in heaven now.” (Interviewee 10).

Rural women imagined a good place in the hereafter for their relatives who had died in the earthquake because they had died from natural disasters. This way, they could calm themselves down.

### Negative interactions

Negative Interactions: Despite some women who found positive ways to cope with the earthquake, some others were not able to react in the same way and chose ways to cope with the earthquake that could make their situation worse and more vulnerable. Negative interactions refer to the negative methods that lead to some kind of inappropriate adaptation to the events after the earthquake.

1. Annoyance at God: After the earthquake, the survivors may feel that God has punished them. Therefore, they get annoyed and sulk.

“I had never missed a single prayer until the earthquake took my everything. So, I have decided not to say my prayers any longer.” (Interviewee 3).

“After the earthquake, I cannot establish a close relationship with God because I feel he doesn’t see us anymore and I feel he likes us to die.” (Interviewee 8).

Since rural women blamed God for what was happening to them, some of them reacted to this by avoiding God and cutting off religious practices.

2. The Phobic Display of a Greater Disaster: After the earthquake, rural women fell victim to a variety of local, national, and international propaganda and rumors, such as HAARP-triggered earthquakes. Therefore, they were obsessing over earthquakes with greater magnitudes.

“I have a gut feeling that a bigger earthquake happens and we all die.” (Interviewee 14).

“I am positive there is going to be a bigger earthquake whereby everyone else will be killed. Every night, I think with myself that there may be another earthquake. So, I can’t sleep a wink all night long.” (Interviewee 6).

Despite warnings from national and local authorities that the HAARP phenomenon was not occurring, advertisements on social networks put more pressure on the earthquake-stricken people, and, in many cases delayed both their return home and starting the life over.

3. Aggression towards the Others: Women are faced with a great deal of stress after the earthquake, possibly creating a kind of violence and aggression towards people around them, especially children.

“I had never hit my 10-year-old child before the earthquake, but I do not know why I lost my temper and slapped him.” (Interviewee 17).

“After the earthquake, I have lost my temper so much that I keep picking on my kids and hit them.” (Interviewee 5).

Having violent behaviors towards others was another reaction of women to the earthquake that could make the situation worse than it was and make their lives more stressful.

4. Isolation: After the earthquake, the psychological and social conditions cause some women to stay away from the others, thus worsening their psychological problems.

“After the earthquake, I am not in the mood to do anything, and I don’t even feel like going out of the container home or seeing anyone.” (Interviewee 22).

“Before the earthquake, I paid visits to my relatives once a week or so, but now, it has been nearly two months I have not gone out of the village. Besides, I don’t leave my house when I am at the village.” (Interviewee 11).

Staying away from one’s community and isolation can have far-reaching consequences for rural women, and if this is not resolved, it can create more psychological problems.

5. Death Wish: The demolition and psychological burden of the earthquake were so extensive in Kermanshah that after the earthquake, some women thought of death all the time and preferred to die. Many rural women could not cope psychologically with the situation, and since there were no suitable programs for their mental rehabilitation, many had no hope of returning to normal life and were thinking of committing suicide, and some even did this.

“I see myself much closer to death than before, and I would like to die.” (Interviewee 16).

“I can’t stand this much stress and pressure any longer. I used to be scared of death, but now I do not care about it anymore. For instance, I have thought of attempting suicide on many occasions, but just because of my family, I haven’t plucked the courage to do it.” (Interviewee 6).

In fact, rural women were getting closer to death because of their hopelessness to improve, and this can make it harder for them to return to life and cope with the issues.

## Discussion

In addition to destroying houses, earthquakes can bring about massive destruction and threaten the lives of many people, especially rural women, as one of the most vulnerable groups in the event of earthquakes. It should be noted that women’s ability to come to terms with natural disasters such as earthquakes relies on how well their needs and concerns are understood by health planners and policymakers [32]. Therefore, examining the needs of women in the context of society and culture is necessary [31].

The results of the present study demonstrated that the health needs of women were neglected in the earthquake, which could endanger their health. When natural disasters occur in third world societies, some of the vital needs are neglected due to the lack of a proper supportive structure, and the same case goes for Kermanshah Province, too. For example, the health needs of women were neglected for months after the earthquake. Similarly, in studies conducted by Nakhaei et al. (2015), Hirth et al. (2013), and Rahmani Bilandi et al. (2015), the neglect of women’s health needs has been reported [31, 33, 34]. Our findings, therefore, add to the already strong body ofevidence showing that for effective disaster response and recovery, there is a need for explicitconsideration of the health needs of women – ideally involving women themselves – at the planningstage.

The results of the present study indicated that earthquakes could cause tensions in families, and since the circumstances of families are extensively affected by earthquakes, it can lead to tensions in marital relationships and disruptions in family functioning [19]. Chan et al. (2011) reported that family violence such as physical and psychological violence against women was increased as a result of earthquakes [24]. Also, the results of a study done by Campbell et al. (2016) revealed that violence against women was increased after the occurrence of earthquakes [23]. Research also has shown that women’s satisfaction with their sexual lives is dramatically decreased [25]. The results of this study, in comparison with the results of previous studies, revealed that more attention should be paid to the family after natural disasters because family is the source of peace, otherwise it will turn into a source of stress and pressure increasing the post-earthquake problems.

Gender inequity in the provision of services and aid was one of the other categories in the present study. Women in rural areas did not have access to resources due to the lack of sufficient literacy and restrictive culture in the village. Additionally, because of the dominant patriarchal culture in these areas, women did not have the opportunity to receive aids. Therefore, the lack of access to vulnerable groups, such as women, to services can impose irreparable damages to them. Research has shown that, compared to men, women’s health is more at risk during disasters due to personal, social, and health-related factors. In other words, the vulnerabilities of women’s health in the events of natural disasters deteriorates because of inequality in facing disasters and access to resources and opportunities [35]. Providing health services in the event of natural disasters based on gender is of prime importance since, in addition to the social and cultural limitations that women face in receiving health services, they have their own health needs, such as those during menstruation, pregnancy and breastfeeding their babies [34].

Feeling insecure was another category in the present study. Since the presence of aid workers and volunteers changed the previous social atmosphere of villages, it created a kind of insecurity in rural women because of their unfamiliarity with the new situation, which made women decide not to go out. It should be noted that there was no report of sexual assault due to the specific cultural context of the target area and the sensitivities that exist in this respect among Kurds.

The results of the present research revealed that the ruling culture of the region had been ignored when the survivors of the earthquake were provided with services. For this reason, most of the delivered services were not used due to the incompatibility of the sent clothing and food with the native culture of the region. For example, many bathrooms were built in villages, but most women did not use them because of the cultural conditions governing their villages. It should be noted that providing services to earthquake victims should be community-based and based on the culture of the region so that the needs of certain groups such as children, women and the elderly to be taken into account [36]. In addition, the values and norms governing the region should be considered in this respect.

Another result of the present research was about the rural women’s unmet needs, especially their health-related needs, due to concealing them for fear of stigmatization. Likewise, in a study done by Rahmani Bilandi et al. (2015), it was reported that indigenous beliefs and cultural taboos were the main reasons for earthquake-stricken women’s concealing their needs, thus exacerbating their health problems [34]. The results of a study conducted by Bahman Jonbeh et al. (2018) revealed that women were reluctant to ask for any health care services such as sanitary pads because of cultural taboos and their shyness [30]. The results of this study added to previous studies that much of the needs of rural women remain unaddressed due to the conditions prevailing in the village, thus considering the social and cultural conditions of the villages as well as providing appropriate opportunities, such as the use of female social workers to assess women’s needs can be a good way for women to express their needs easily.

The incoherent mourning was another category in the present study. Those who lose their relatives during an earthquake suffer from the loss of their loved ones for a long time because they cannot mourn their deaths properly.

One of the issues that matter after earthquakes is adaptability and coping with emergencies afterward. Some people have more adaptability and less vulnerability whereas there are some who cannot cope with this problem and are more exposed to damages. Adaptability and coping are the topics of interest to researchers who study the effects of natural disasters on communities [37, 38]. Adaptability after such terrible disasters is affected by various factors, such as personal ability, religious beliefs and faith and external support [39, 40]. Not to mention, women are more susceptible to disasters and their compatibility capacity is reduced under the effects of gender roles, norms and unequal access to power and resources [41]. The findings demonstrates that the survivors were upset about the process of the funeral ceremony for their loved ones and they had a guilty conscience was found for the first time in this study; therefore, when authorities bury the dead after natural disasters, they have to maintain their dignity so that their families do not feel bad.

The results of the present study revealed that rural women’s reactions to earthquake fell into two categories: positive and negative interactions. In the former, they approached this phenomenon by getting closer to God, participating in group works, comparing their current circumstances with other worse conditions, sharing feelings and experiences, and heroizing the dead.

Getting closer to God through religious practices and prayers was one of the strategies that rural women adopted to come to terms with the earthquake, which created a kind of inner satisfaction in them. Likewise, the results of previous studies showed that religious practices were recognized as one of the strategies for coping with natural disasters [42–44].

Participation in group works was another strategy adopted by rural women to come to terms with the earthquake and to make them too occupied with group works in the community to think about problems, thus having less stress.

Another category of positive interactions among earthquake-stricken women was comparing their own circumstances with others’ worse conditions. Since earthquakes can lead to loss of many lives, everyone is exposed to this problem, and the majority of victims compare their problems with those of others to relieve their pain and psychological burden.

Sharing of memories was another way whereby rural women could come to terms with the earthquake consequences. After the earthquake, rural women gathered around their tents and container homes and shared the memories of the night of the earthquake, thus encouraging them and reducing their post-earthquake stress [45]. Similarly, the results of a study done by Ahmadi et al. (2018) revealed that sharing emotions and experiences with neighbors was one of the strategies to come to terms with the earthquake consequences [42]. In fact, this strategy can be used as a kind of local social capital to play a positive role in compatibility with natural disasters [45, 46].

One of the other ways that rural women came to terms with the death of their loved ones was to heroize them, and they were believing that their relatives would have God’s mercy on their souls because they lost their lives in the earthquake. On the other hand, having found themselves in tremendously difficult conditions, they supposed that the dead were finally relieved and adored by God too much to let them experience intolerable conditions. However, some rural women found it hard to come to terms with the consequences of earthquakes, and their reactions included annoyance at God, phobic display of a greater disaster, aggression towards the others, isolation, and death wish.

Since earthquake is seen as something that occurs with the will of the Almighty God, its occurrence in some cases changes the relationship between the survivors and God, and because the earthquakes are regarded as a punishment by God, they find it an annoyance.

The phobic display of a greater disaster was another issue that was obtained from the data. In the area under study, after the major earthquake, there were a lot of aftershocks that exposed people to high levels of mental pressure to the extent that the mental pressure of aftershocks was more irresistible than that of felt during the main earthquake itself. In the meantime, rural women fell victim to a variety of local, national and international propaganda and rumors, such as man-made earthquakes, due to insufficient media literacy. They also fell for rumors that there would be more destructive earthquakes at any time. Accordingly, they were obsessing over worse happenings. Consistent with the findings of the present study, previous studies revealed that worry and fear after a natural disaster were more common among female survivors than men [47].

Aggression towards other people was another result of rural women’s negative interactions because they experienced a lot of stress after the earthquakes, thereby leading to aggression and violence. This finding was concurrent with the results of studies in which it was reported that aggression and violence were prevalent in the families after earthquakes [19, 24].

Isolation was another alternative taken by rural women after the earthquakes, thereby excessively increasing their psychological burden and making it less likely to appear in community and to express in their desires and needs.

Death wish was another category in the present study. The earthquake that occurred in Kermanshah destroyed lots of residential buildings and the subsequent damages led to psychological problems and hopelessness in women. Furthermore, since rural women in this area had little knowledge to deal with critical conditions, they got distressed and desperate, and thought of death and suicide. For example, in a study conducted by Hyodo et al. (2010) on the victims of an earthquake in Japan, the tendency towards death and suicide was on the rise among women [48]. Likewise, Yang et al. (2005) and Yip (2009) concluded the same finding among the earthquake survivors [49, 50]. The findings of this study add this point to previous studies that, after the earthquake, some women deprived themselves from all the services they were provided with and this could worsen their condition. So, paying more attention to these women and providing conditions for them to be more present in society can be effective in improving their health faster.

## Conclusion

The rural women, troubled by earthquakes are usually benefited from the fewest facilities and always in need of health and financial basics, which are affected by their social and cultural conditions, such as gender inequity in the provision of assistance, concealing needs for fear of stigmatization and ignoring the ruling culture in the area. Additionally, the results of the present study revealed that rural women’s reactions to earthquake fell into two categories: positive and negative interactions. In the former, they approached this phenomenon by getting closer to God, participating in group works, comparing their current circumstances with other worse conditions, sharing feelings and experiences, and heroizing the dead. However, most rural women found it hard to adopt such type of positive adjustment and negatively reacted to the consequences of earthquakes, including annoyance at the God, phobic display of a greater disaster, aggression towards the others, isolation, and death wish. Hence, in the event of natural disasters, such as earthquakes, paying more attention to the needs of rural women, taking the culture governing the village into account at the time of service delivery and helping them with positive adaptations are some indispensable measures that should be taken.

## Data Availability

Data are available by contacting the corresponding author.
